# An Introduction to the Analysis of Single-Cell RNA-Sequencing Data

**DOI:** 10.1016/j.omtm.2018.07.003

**Published:** 2018-08-02

**Authors:** Aisha A. AlJanahi, Mark Danielsen, Cynthia E. Dunbar

**Affiliations:** 1 Translational Stem Cell Biology Branch, NHLBI, NIH, Bethesda, MD, USA; 2 Department of Biochemistry and Molecular & Cellular Biology, Georgetown University Medical Center, Washington, DC, USA

**Keywords:** single-cell gene expression, RNA sequencing, computational pipeline, microfluidics, drop-seq, sci-seq, principle component analysis, t-distributed stochastic neighbor embedding

## Abstract

The recent development of single-cell RNA sequencing has deepened our understanding of the cell as a functional unit, providing new insights based on gene expression profiles of hundreds to hundreds of thousands of individual cells, and revealing new populations of cells with distinct gene expression profiles previously hidden within analyses of gene expression performed on bulk cell populations. However, appropriate analysis and utilization of the massive amounts of data generated from single-cell RNA sequencing experiments are challenging and require an understanding of the experimental and computational pathways taken between preparation of input cells and output of interpretable data. In this review, we will discuss the basic principles of these new technologies, focusing on concepts important in the analysis of single-cell RNA-sequencing data. Specifically, we summarize approaches to quality-control measures for determination of which single cells to include for further examination, methods of data normalization and scaling to overcome the relatively inefficient capture rate of mRNA from each cell, and clustering and visualization algorithms used for dimensional reduction of the data to a two-dimensional plot.

## Main Text

Until recently, single-cell gene expression profiling was limited to studying several select transcripts from a few individual cells. High-throughput sequencing along with high-yield cell separation methods have paved the way to modern single-cell sequencing platforms such as Fluidigm C1, DropSeq, Chromium 10X, SCI-Seq, and many others that have been developed over the past decade (Table 1). These technologies are able to characterize the transcriptional profile of hundreds up to many thousands of single cells at a time. All rely on labeling mRNA molecules with DNA barcodes during reverse transcription and/or subsequent steps, which allows indexing of the transcripts back to their individual cells of origin. Although each method is unique in the way it separates cells and labels the mRNA molecules, they all rely on similar computational pipelines for the representation of the transcriptional profiles. In this review, we will discuss some of the most common algorithms used in these computational pipelines, using DropSeq as a primary example, because it is the most cost-effective and widely available single-cell gene expression platform (Table 1). However, these concepts are applicable to most single-cell sequencing platforms that use DNA barcodes as an approach to link mRNA transcripts to a single cell of origin.

**Table 1:** Widely Used Single-Cell Sequencing Methods

*Sequencing Method*	Starting Cell No.	Cell Separation	Notes	Cell Capture	Transcript Capture	Representative Library Prep Cost per Cell^a^
*Fluidigm C1*^b^	∼1,000 cells	cells capture in size-specific chambers	must know the size of cells of interest; allows for staining and imaging prior to cell rupture	96- or 800-chamber units are available	an average of 6,606 genes/cell (no data on percentage)	$1.70
*DropSeq*	∼150,000 cells/run	droplet-based separation	remains the most cost-effective and most customizable	∼5% of cells per run (approximately 7,000 cells)	∼10.7% of the cell’s transcripts	$0.06
*Chromium 10X*	∼1,700 cells/run	droplet-based separation	the most commercially successful method; almost fully automated	∼65% of cells per run (approximately 1,000 cells)	∼14% of the cell’s transcripts	$0.10
*SCI-Seq*	∼500,000 cells (depends on experimental design)	FACS sorter; cells are never singly isolated	combinatorial indexing of individual methanol-fixed permeable cells	5%–10% of cells	∼10%–15% of the cell’s transcripts	$0.05–$0.14^c^

All of the methods require the establishment of a cell dissociation technique. The price is highly dependent on the number of cells sequenced, the desired depth of sequencing, and the sequencing platform used. For this table, the prices are at the lower end of the price range for single-cell library prep.

a As of July 2018.

b Based on the 800-chamber medium-size isolation unit.

c Dependent on how many cells are prepped for sequencing and how many doublets are tolerated.

Single-cell RNA sequencing has been uniquely valuable to gain insights into cellular heterogeneity in tissues and for identification of previously unknown cell types.^1^, ^2^, ^3^ Single-cell technologies can also be used to define subpopulations within a known cell type by searching for differential gene expression patterns within the cell population of interest.^1^, ^4^ In addition, these technologies can effectively isolate the signal from rare cell populations, which would be hidden in output from bulk cell population RNA sequencing.^5^, ^6^, ^7^, ^8^ Moreover, the technology can be used to infer potentially useful markers, such as cell surface proteins, for cell types with no known markers. Because single-cell sequencing analysis is driven by clustering of cells based on their differentially expressed genes, the genes that drive the clustering can be examined as possible unique markers for the cell population of interest.^1^, ^9^ Lastly, single-cell sequencing can be employed in studies of cell lineage and the regulation of differentiation. For example, a population of stem cells can be induced to differentiate, and single-cell sequencing performed at series of time points can provide “snapshots” of the progression of differentiation. These snapshots can then be used to infer the trajectories that cells follow to reach each terminally differentiated state and the key genes that are differentially regulated at each branch point.^1^, ^10^, ^11^, ^12^

Many of these applications rely on specialized algorithms that have been developed and made available by leading bioinformatics labs. However, in this review, we will focus on the basic quality-control and data normalization pipeline that all single-cell sequences must undergo before applying any specialized algorithms. We will also discuss simple cell clustering and visualization algorithms.

### Generation of Single-Cell Expression Datasets via Droplet Methodologies

**Figure 1:**
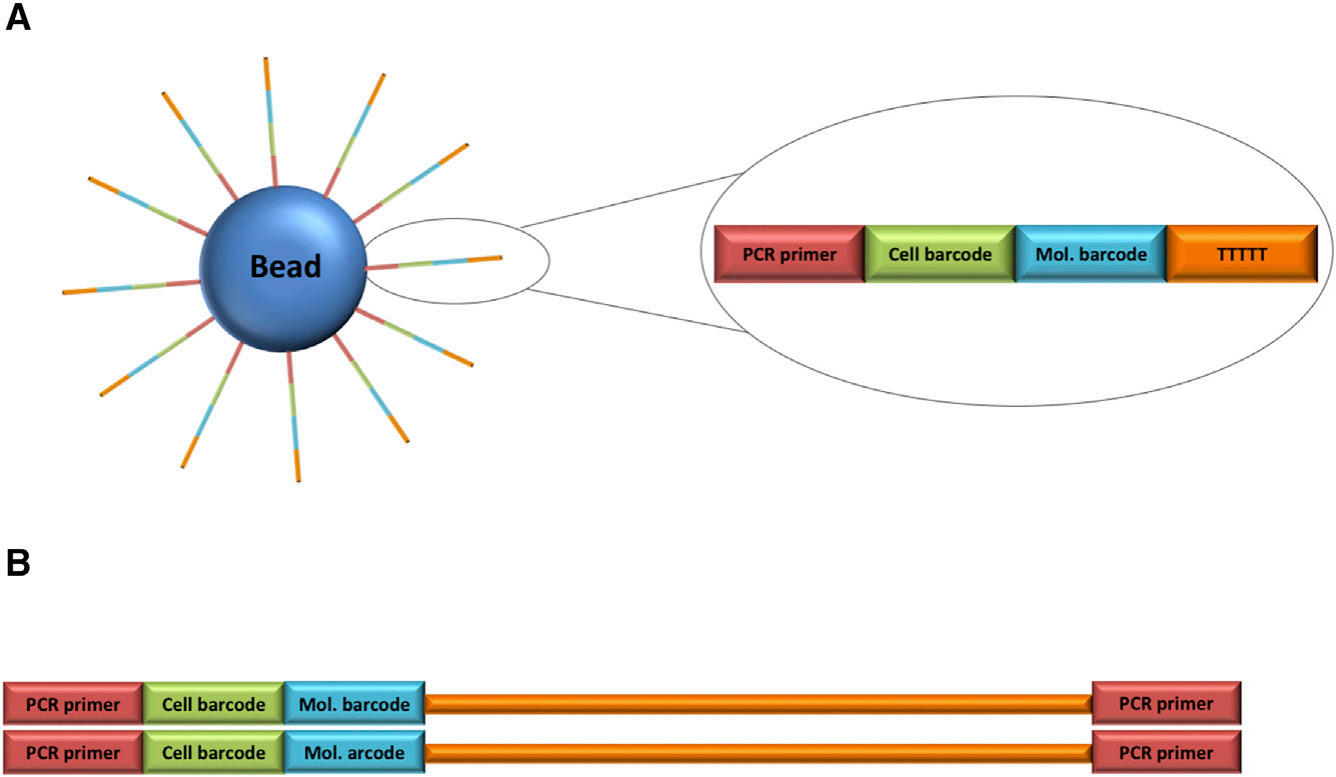
The Structure of Drop-Seq Bead and Resulting Sequence Libraries (A) The structure of a DropSeq single-cell sequencing bead. The oligos extending from the bead have a PCR primer, a cell barcode that is unique to the bead to label each cell, a UMI that is unique to each individual oligo arm to allow unique labeling of each captured molecule, and a poly(T) tail to capture poly(A)-tailed mRNAs. (B) Structure of the sequencing ready library. Red: PCR primers which are also used as sequencing primers. Green and blue: the cellular and molecular barcodes from the bead. Orange: the captured transcript with the poly(A/T) tail.

Droplet-based single-cell gene expression approaches, including DropSeq^13^ and the commercial 10X platform,^14^, ^15^, ^16^, ^17^, ^18^, ^19^ use microfluidic chips to isolate single cells along with single beads in oil-encapsulated droplets, using microfluidics to bring oil, beads, and cell suspensions together in such a way that each droplet contains at most a single cell.^20^ The beads are coated with DNA oligos that are composed of a poly(T) tail at the 3′ end for the capture of cellular mRNAs, and at the 5′ end both a cell barcode that is identical for every oligo coating an individual bead and a library of individual unique molecular identifier (UMI) barcodes of high diversity, each UMI different for every oligo on the bead (Figure 1A).^13^, ^21^, ^22^ The transcripts from each individual cell captured and labeled by the DNA oligos attached to a bead within the droplets are reverse transcribed, amplified with PCR, and sequenced using a high-throughput platform, after breaking and pooling droplet contents.^23^, ^24^, ^25^, ^26^, ^27^, ^28^ The resulting sequences are aligned to a reference genome in order to annotate each transcript with its gene name. The cell barcodes on the aligned sequences allow for the computational linking of each gene transcript to its cell of origin. The number of copies of individual gene transcripts expressed in each individual cell is tallied using the UMIs, allowing the assembly of digital gene expression matrices (DGEs), which are tables of cell barcodes and gene counts.^13^, ^21^, ^29^, ^30^, ^31^

The insights feasible from such complicated sequencing data are only as good as the computational interpretation that follows. Most available single-cell sequencing algorithms do not have a graphic user interface. Thus, performing single-cell analysis requires knowledge of some programming languages in order to interact with the pre-established algorithms for aligning, clustering, and visualizing the data. In addition, in-depth knowledge of the biology of the cells of interest is essential to correctly interpret the data and make appropriate decisions based on quality-control measures. A bioinformatician with expertise in single-cell sequencing is able to generate analyses that can be used to make meaningful biological inferences, choosing appropriate cutoffs for the algorithms applied and avoiding misleading results.

### Quality-Control Metrics

**Figure 2:**
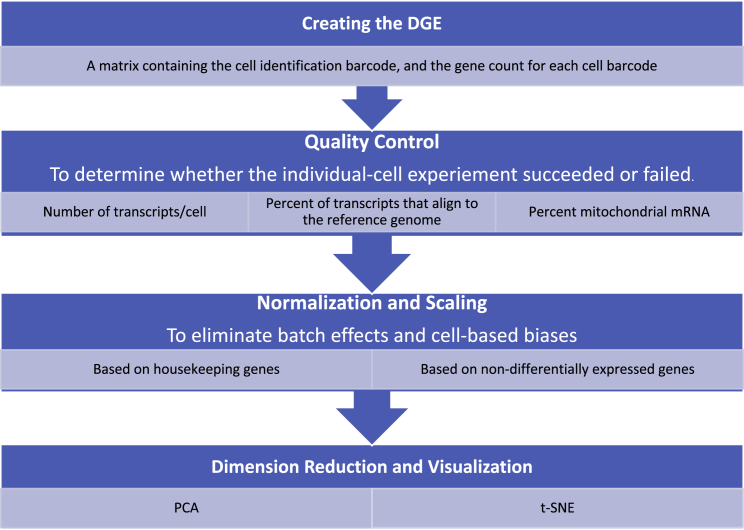
Analysis Workflow

Because droplet-based experiments can be considered to be thousands of separate experiments taking place on individual cells within individual droplets, it is essential to apply quality-control (QC) metrics designed to decide which of these individual droplet datasets is valid for further interpretation (Figure 2). QC can most effectively be performed on droplet-based datasets by applying a number of different parameters that detect unsuccessful droplets and exclude their data from further analyses.^13^, ^32^, ^33^, ^34^, ^35^, ^36^, ^37^

QC parameters are unique to each run because they are dependent on the cells or tissue being sequenced. A common QC metric is the number of transcripts tallied per cell, or the percent of transcripts per cell that align to the reference genome. Cells with transcript counts below or above a defined cutoff are marked as outliers and are not included in further analysis; these outlier cutoffs can be user-defined for each experiment (e.g., cells with less than 20 transcripts and more than 5,000 are removed from the analysis), or automatically applied by the program (e.g., cells with a sum of transcripts larger than 2 SDs from the mean are removed). The presence of a very large number of transcripts with one cell barcode may result from doublets (i.e., two or more cells suspended in one droplet), and such data are eliminated from the analysis. Conversely, a small number of transcripts per cell barcode is often an indicator of poor capture quality, which could be because of cell death, premature cell rupture, or the capture of random mRNA escaping from cells and floating in the cell suspension reagents.^13^, ^32^, ^34^, ^36^, ^37^ Additional QC metrics can be applied, for instance, simply excluding all cells that express a specific gene to remove contaminating cells that are not of interest from an analysis, or can be more elaborate, for example, including only cells that have a specific ratio of two or more specific genes.^33^, ^36^

When deciding on QC cutoffs, the diversity of the tissue being analyzed must be taken into account. For instance, when designing an experiment to study migrating cancer cells found in the blood, where the number of cancer cells is very low compared with the overall number of normal blood cells, the *counts of transcripts* QC metric must be adjusted. In this tissue, the dominant cells are blood cells, which are generally quiescent and have relatively low amounts of RNA compared with active cancer cells.^38^ If all cells with a transcript count higher than 2 SDs from the mean are removed from the analysis, it could lead to the elimination of all cancer cells, mistaking them for doublets because of their high transcriptional activity compared with the much larger population of blood cells. Setting cutoffs appropriately may require spike in experiments prior to running experimental samples.

Another common QC metric is the number of mitochondrial gene transcripts.^32^, ^33^, ^35^, ^39^, ^40^ High numbers of mitochondrial transcripts are indicators of cell stress,^41^ and therefore cells with elevated mitochondrial gene expression are often not included in the analysis, because most experiments will not benefit from clustering cells based on stress levels. However, just as with *number of transcripts*, this parameter is highly dependent on the tissue type and the questions being investigated. For example, 30% of total mRNA in the heart is mitochondrial due to high energy needs of cardiomyocytes, compared with 5% or less in tissues with low energy demands.^42^ For instance, 30% mitochondrial mRNA is representative of a healthy heart muscle cell, but would represent a stressed lymphocyte.

Depending on the goal of an experiment, a gene-specific QC metric can be added. Genes that are always present in very low quantities, and will never reach statistical significance between cell types, may be removed from the analysis to decrease the computational load.^32^, ^33^, ^36^ This can be done by either setting a cutoff of gene count per cell (e.g., gene count in cell = <5 in all of the sequenced cells) or setting a cutoff for the count sum across all or a subset of cells (e.g., ∑count of gene across all of the sequenced cells = <300). Excluding such genes from the analysis will speed the computational process; however, some genes that are very slightly differentially expressed and contribute to the variance of the data might be lost.

### Data Normalization and Scaling

When analyzing sequencing data, normalization to eliminate batch effects is crucial if multiple sequencing runs are to be compared with each other. These batch effects can be caused by often unavoidable technical variations such as the duration samples were kept on ice, number of freeze-thaw cycles, method of RNA isolation, sequencing depth, etc.^43^, ^44^, ^45^ Investigators should always strive to keep these variables as constant as possible between experiments and sequencing runs. However, droplet-based sequencing in addition consists of thousands of individual cell experiments, hence cell-specific biases must also be considered when normalizing, in order to be able to compare the expression of one cell to another.^46^, ^47^ A notable cell-specific bias is caused by mRNA capture efficiency, where the mRNA molecules are not captured by the bead at the same proportion in all droplets (Table 1). This is referred to as “dropout events,” and it is the main cause for sparsity of data, which we will discuss further in the next paragraph. Furthermore, for bulk RNA sequencing, normalizing data involve comparing multiple batches of similar biological material (e.g., comparing blood cells with blood cells), but in single-cell sequencing the individual cells are not all of the same type. This requires adjusting the normalization parameters to retain cell-to-cell variability while eliminating technical noise caused by batch effects and cell-specific biases.^47^

The sparsity of data, due to the inefficiency of mRNA capture (i.e., at best, DropSeq is predicted to capture about 10% of each cell’s mRNA^13^) poses the biggest challenge for the analysis of droplet single-cell sequencing data. The DGE matrix is expected to be mostly filled with zeros because of these dropout events.^48^ Therefore, normalization and scaling are vital prior to interpreting the data.^44^ Unfortunately, this requires making assumptions about the cells that can be biologically inaccurate. An accepted way to normalize the sequencing data is based on comparisons with housekeeping genes.^32^ Based on literature and knowledge of the biological sample sequenced, a housekeeping gene is selected for normalization. The selected gene is assumed to be expressed at the same level in all cells, and the sequencing data are scaled to make the expression level of the selected housekeeping gene equal in all cells. However, the housekeeping gene method can be inaccurate because these genes are not always present in the same amount in different cell populations.^49^, ^50^ To avoid making the assumption that a housekeeping gene is present in an equal amount in all cells, the scaling can be based on all non-differentially expressed genes in all or some of the cells.^32^, ^51^ This approach assumes that all genes that are non-differentially expressed between cells are expressed equally in all cells, and it infers a scaling factor for each cell that is used to normalize transcript counts.^32^

### Dimension Reduction and Visualization

**Figure 3:**
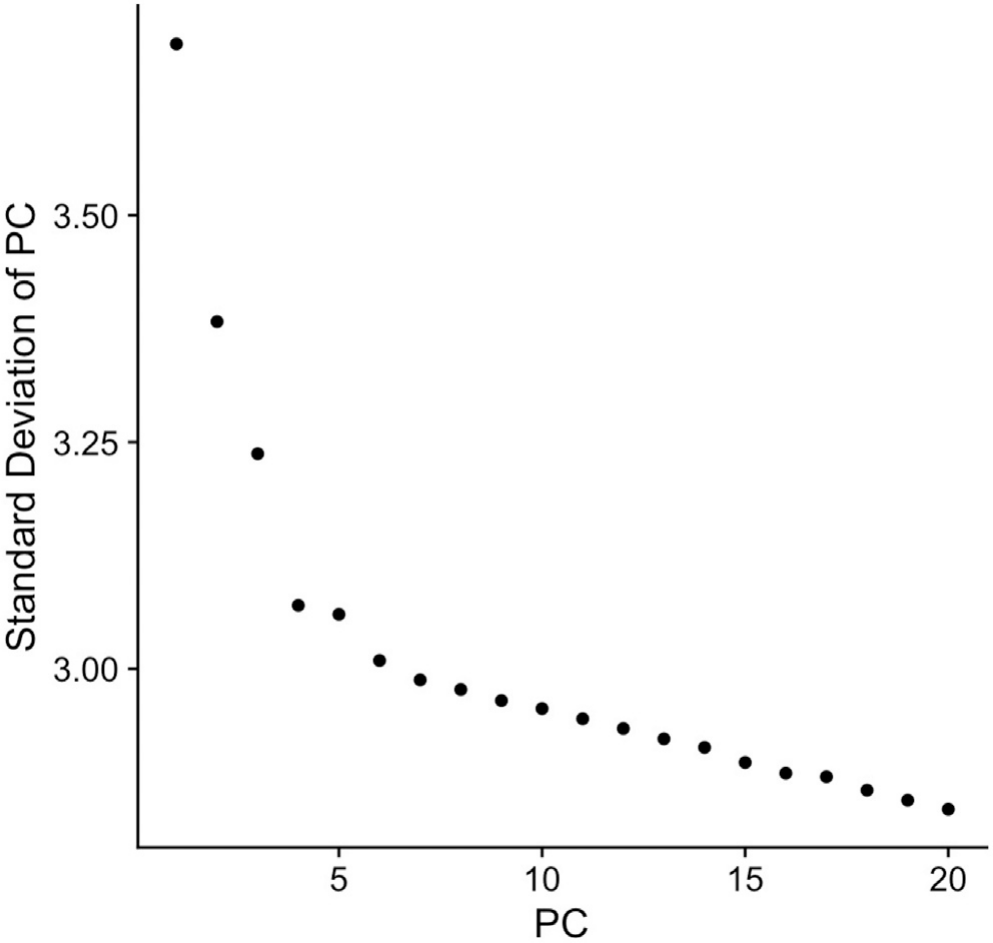
Elbow Plot Analysis of Principle Components Variance A plot of the SD of each principal component, representing the amount of variance it contributes to the data. Here, the plot shows an elbow at around PC5. PC4 and PC6 are also valid choices for the PC cutoff.

After normalization, an unbiased clustering algorithm can be used to determine which cells are closely related based on their gene expression profiles. Principal component analysis (PCA) is often the clustering algorithm of choice, because it is a relatively simple linear dimensionality reduction algorithm that can predict the relatedness of multidimensional data, or in this case, predict the relatedness of cells based solely on differential gene expression.^5^, ^10^, ^32^, ^37^, ^52^, ^53^, ^54^, ^55^, ^56^ PCA merges the information from correlated genes into one “metagene” or principal component (PC). By definition, PC1 explains the greatest possible variance in the data and has the largest SD (e.g., for a specific experiment 30% of the variance between the cells is explained by genes that define PC1). PC2 explains the second greatest portion of variance in the data, and so on (e.g., an additional 20% of the variance between the cells will be due to genes that define PC2, and an extra 8% is attributed to the genes that define PC3). The PCs are ranked based on significance of explaining the data’s variance, with PC1 being the highest-ranking PC. The lower ranking the PC, the less it contributes to explaining the variance of the data. Therefore, using the lower ranked PCs is generally not advantageous because it increases the computational load, yet barely adds any information to the representation of the biological variability of the cells. Thus, deciding how many PCs to use for visualization is important. This can be done using visual plotting methods. A simple example is the knee or elbow plot like the one shown in Figure 3, where the SD for each PC is plotted to represent the amount of variance encompassed within that PC.^37^ As expected, the first PC contributes the most to the variance and has the highest SD. After the fifth PC, the contribution to the explanation of variance plateaus. That is where the “elbow” is determined to be, and it becomes the cutoff for PC inclusion in visualization.

t-Distributed stochastic neighbor embedding (t-SNE) is a common visualization approach.^57^, ^58^, ^59^ It uses a machine learning algorithm that reduces dimensions and is well suited for embedding high-dimensional data into two- or three-dimensional space for visualization, without losing information about the relative distance between the plotted data points or, in this case, cells. For example, if the diversity of the cells was found to be well represented with seven PCs, then seven axes or dimensions are required to represent the cells. t-SNE will plot the cells on a two-dimensional plot in a way that maintains the seven-dimension relationship between cells, so that cells that are neighbors on a seven-dimension plot remain neighbors on a two-dimension plot. Whereas PCs analysis is linear, t-SNE is a non-linear dimensional reduction method.

### Considerations Regarding Data Generation Efficiency and Alternative Single-Cell Platforms

The computational approaches discussed in this review are focused on droplet-based separation methods such as DropSeq and Chromium 10X. However, because most single-cell sequencing platforms share the main principal of labeling mRNA from each cell with unique DNA barcodes, then computationally tracing back these molecules to their cells of origin, similar principles and algorithms can be used for datasets from other approaches, keeping in mind the type of technical variations or artifacts that may result from specific processes included in different platforms. These platforms and methods of cell separation, labeling, and DNA amplification have been recently reviewed in detail by Valihrach et al.,^60^ where they discuss the basic principles of each platform and the advantages and pitfalls of the methods.

For example, in SCI-Seq,^61^ cells are fixed with alcohol, making them permeable. The fixed cells are sorted using a flow cytometric sorter, dispensing specific numbers of cells into each well on a multiwall plate (Table 1). The mRNA of the cells in each well is barcoded with an oligo unique to that well via reverse transcription. The cells from all wells are then pooled, and another round of fluorescence-activated cell sorting (FACS) of cells into wells at lower density is performed, followed by adding a second unique well-specific barcode, creating a unique barcode combination for each cell. This process can be repeated again to reduce the chance of two cells being labeled with the same barcode combinations. This combinatorial approach to single-cell labeling requires a specialized algorithm to create the DGE matrix, because in contrast with droplet-based methods, a single cell is not defined by a single barcode but instead by a unique combination of barcodes. It is worth noting that this approach, requiring at least two rounds of cell sorting, could be more stressful to cells and impact on gene expression.

Another example is the interpretation of the *number of transcripts/cell* parameter to exclude doublets, because each method is expected to produce a different rate of doublets. In the Fluidigm C1 system, where individual cells are captured by size-specific chambers, the doublet rate drops from 7% to 3% after microscopic examination of cells in the 96-chamber medium size isolation unit (Table 1).^62^ The rate is not zero because cells are sometimes stacked on top of each other in the isolation chamber, making them look like single cells, and therefore can be missed by microscopy.^63^ If the number of transcripts is significantly higher (e.g., more than 2 SDs higher than the mean) in more than 3% of the microscopically examined cells or 7% of non-examined cells, this could indicate a mixed cell population composed of a small fraction of transcriptionally active cells and a larger portion of quiescent cells, or it could be due to a high rate of true doublets, in which case the size of the size-specific isolation chambers might be inappropriate for the cell population being studied.

It should be noted that most popular single-cell analysis pipelines are driven by the most differentially expressed genes between cells. This is beneficial for finding gene markers for unknown populations.^64^, ^65^, ^66^ However, if researchers aim to study cell types that are very similar, or find subpopulations within one major cell type, then those cells can be sorted prior to analysis in order to increase the number of cells of interest, thereby increasing the power of the analysis. Even though FACS has been shown to have a minimal effect on gene expression,^67^, ^68^ sorting prolongs the time that cells are not in optimal culture conditions and kept in a single-cell suspension, which could stress the cells and possibly alter mRNA and mitochondrial mRNA expression.^68^, ^69^, ^70^, ^71^ Also, passing of cells in small chambers, or through microfluidics or a cell sorter can cause shear stress and impact some cell types more than others in terms of causing cell stress or death, especially because the cells are vulnerable in a single-cell suspension.^72^, ^73^, ^74^ Therefore, delicate cell types might be under-represented in droplet-based single-cell sequencing experiments, especially if the cells were sorted prior to single-cell isolation.

### Conclusions

In summary, we have discussed concepts important to applying analytic pipelines for the analysis of single-cell gene expression data, and specific parameters that change depending on cell types or condition. We also provide examples of some types of technical variation that need to be considered in order to adapt this pipeline to non-droplet-based methods. The pipeline starts with the creation of a DGE matrix, which contains gene counts in each cell, from the raw sequencing files. The rest of the analysis is applied on this matrix file. QC determines which cells to exclude from downstream analysis because of various reasons like the suspicion of doublets or cellular stress. Normalization and scaling are then performed to compensate for the sparsity of data because of the low mRNA capture rate. Then, dimension reduction is done based on the most differentially expressed genes. Finally, if done correctly, visualization of the data will result in plots showing the relatedness of each cell to its neighbor in two- or three-dimensional space.

These algorithms are in general use and are often included in an easy-to-use packages such as Seurat^13^, ^37^ (https://satijalab.org/seurat/), an R-based package that creates R objects compatible with other downstream algorithms; scran^51^ (http://bioconductor.org/packages/release/bioc/html/scran.html), which also includes algorithms for cell-cycle assignment; ascend^75^ (https://github.com/IMB-Computational-Genomics-Lab/ascend), which includes well-established and new algorithms providing a flexible analysis framework; and many others.^33^, ^34^, ^36^, ^37^, ^46^, ^76^, ^77^, ^78^, ^79^, ^80^, ^81^, ^82^, ^83^, ^84^, ^85^, ^86^ A few of these packages were reviewed by Yip et al.^87^ comparing their accuracy and precision in detecting highly variable genes. These pipelines are usually followed by more specialized algorithms, depending on the purpose of the experiment. A few examples of specialized algorithms include Monocle (http://cole-trapnell-lab.github.io/monocle-release/), an algorithm designed to analyze differentiation trajectories of single cells;^11^, ^12^ SingleSplice (https://github.com/jw156605/SingleSplice), used to study alternative splicing in single-cell populations;^88^ and OncoNEM (https://bitbucket.org/edith_ross/onconem/src), a tool to infer the hierarchical evolutionary relationships between tumor cells based on their somatic mutations.^89^ Extensive collections of single-cell sequencing tools and their applications can be found on websites such as https://github.com/seandavi/awesome-single-cell and https://www.scrna-tools.org/, which are updated frequently as new tools become available.

## Conflicts of Interest

The authors have no relevant conflicts of interest to report.
